# Expression of Panton-Valentine Leukocidin mRNA among *Staphylococcus aureus* Isolates Associates with Specific Clinical Presentations

**DOI:** 10.1371/journal.pone.0083368

**Published:** 2013-12-12

**Authors:** Fangyou Yu, Ying Liu, Yuanyuan Xu, Yongpeng Shang, Danping Lou, Zhiqiang Qin, Chris Parsons, Wu Zhou, Xiaoying Huang, Yuping Li, Longhua Hu, Liangxing Wang

**Affiliations:** 1 Department of Clinical Laboratory, The First Affiliated Hospital of Wenzhou, Medical University, Wenzhou, Zhejiang, China; 2 Department of Respiratory Medicine, The First Affiliated Hospital of Wenzhou Medical University, Wenzhou, Zhejiang, China; 3 Departments of Medicine and Microbiology, Louisiana State University Health Sciences Center, New Orleans, Louisiana, United States of America; 4 Department of Clinical Laboratory, The Second Affiliated Hospital of Nanchang University, Nanchang, Jiangxi, China; Rockefeller University, United States of America

## Abstract

Panton-Valentine leukocidin (PVL; gene designation *lukF/S-PV*) is likely an important virulence factor for *Staphylococcus aureus* (*S. aureus*), as qualitative expression of the protein correlates with severity for specific clinical presentations, including skin and soft tissue infections (SSTIs). Development of genetic approaches for risk-assessment of patients with *S. aureus* infections may prove clinically useful, and whether *lukF/S-PV* gene expression correlates with specific clinical presentations for *S. aureus* has been largely unexplored. In the present study, we quantified *lukS-PV* mRNA among 96 *S. aureus* isolates to determine whether expression levels correlated with specific clinical presentations in adults and children. Expression level of *lukS-PV* mRNA among isolates from skin and soft tissue infections (SSTIs) was significantly greater than among isolates from blood stream infection (BSIs), and expression level of *lukS-PV* mRNA among BSI isolates from children was significantly greater than for BSI isolates among adults. Moreover, expression level of *lukS-PV* mRNA among community-acquired (CA) isolates was significantly greater than for hospital-acquired (HA) isolates. These data justify additional studies to determine the potential clinical utility for *lukS-PV* mRNA quantification as a predictive tool for severity of *S. aureus* infection.

## Introduction

*S. aureus* causes a range of clinical infections and presentations, from mild superficial skin infection to life-threatening disease[1]. *S. aureus* infections, especially those caused by methicillin-resistant *S. aureus* (MRSA), are common in hospitals and other healthcare facilities. Highly virulent community-associated MRSA (CA-MRSA) strains emerged in the mid-1990s and are responsible for severe infections in individuals lacking health care-associated risk factors[1,2]. The S. *aureus* virulence factor known as Panton-Valentine leukocidin (PVL; gene designation *lukF/S-PV*) has been implicated in life-threatening presentations caused by CA-MRSA, including severe skin and soft tissue infections (SSTIs) and necrotizing pneumonia[3–5], possibly through induction of cytotoxicity for neutrophils[6]. However, contrasting reports have not found correlations between PVL expression and *S. aureus* pathogenesis[7–9]. A recent meta-analysis revealed that PVL genes are consistently associated with SSTIs, but are expressed only rarely in isolates from more invasive infections[10]. Therefore, the role of PVL in *S. aureus* infections remains somewhat unclear. Enhanced production of PVL in *S. aureus* clinical isolates was associated with less favorable outcomes in a murine skin infection model, where the quantity of PVL production among MRSA and MSSA isolates, while highly variable, remained associated with virulence[11]. Despite strain-to-strain variation, PVL is produced by all *S. aureus* isolates carrying *lukF/S-PV* [12], but whether quantitative expression of *lukF/S-PV* correlates with specific clinical scenarios is unknown. Therefore, the present study was designed to determine whether levels for *lukF/S-PV* expression, quantified as *lukS-PV* mRNA, correlate with specific clinical presentations for *S. aureus* infections. 

## Materials and Methods

### Characterization of *S. aureus* isolates

96 isolates positive for *lukS/F-PV* were recovered from 67 adult and 29 pediatric patients with *S. aureus* infections between January 2003 to December 2010 at First Affiliated Hospital of Wenzhou Medical College, Lishui Central Hospital, the Second Affiliated Hospital of Nanchang University, and Jiangxi Provincial Children’s Hospital. Of the 96 isolates, 54 were associated with bloodstream infections (BSIs), 33 associated with SSTIs, and 9 associated with other infections. Community-acquired (CA) isolates were defined by their isolation within 48 hours after hospital admission, with hosts having no risk factors for nosocomial acquisition and no hospitalizations or nursing home residence within a year before hospital admission. Hospital-acquired (HA) isolates were defined by their isolation more than 48 hours after hospital admission, and from hosts harboring no infections before hospital admission. 58 and 28 isolates were classified as HA and CA, respectively, based on medical records. The remaining 10 isolates could not be classified. All isolates were confirmed as *S. aureus* using a Staph SPA agglutination kit (bioMe'rieux,Marcy l'Etoile, France), Gram stain, coagulase agglutination test, and a Vitek-60 microbiology analyser (bioMe'rieu, Marcy l' Etoile, France). MRSA was confirmed using PCR for detection of *mecA*[13]. The Ethics Committee of the first Affiliated Hospital of Wenzhou Medical College exempted this study from review because the present study focused on bacteria.

### DNA extraction

*S. aureus* isolates were cultured on blood agar (Oxoid, United Kingdom) overnight at 35°C. Three to four bacterial colonies were suspended in 150 μl sterile distilled water with lysostaphin (1 mg/ml) (Sangon, China) and incubated at 37 °C without shaking for an hour. DNA was extracted following the instructions of the Genomic DNA Extraction kit (Sangon, China). The DNA was stored at -20°C and prepared for PCR detection.

### Identification of MRSA isolates and PVL detection

A multiplex PCR protocol described previously was used for simultaneous amplification of *mecA*, 16S rRNA, and *lukS/F* genes[13]. MRSA N315 and *lukS-PV*-positive MRSA isolates identified in our previous study were used as positive control strains[14]. The S. *aureus* isolates positive for *lukS-PV* as determined by the multiplex PCR mentioned above were re-detected using a simplex PCR with specific primers of the multiplex PCR above for *lukS-PV*. The expected DNA products were purified using PCR Production Purification kit (Sangon, China) sequenced by Shanghai Sangon Biotech Ltd.. DNA sequences were assessed by performing a similarity search by BLAST program (Blastn) available in the National Centre for Biotechnology Information (NCBI) (http://blast.ncbi.nlm.nih.gov/Blast). 

### RNA extraction and RT-PCR

All solutions used for RNA extraction were stored at 4°C, and centrifugation steps were performed at 4°C to limit RNase activity. *S. aureus* isolates were cultured in brain-heart infusion (BHI) (Oxoid, United Kingdom) broth overnight at 37°C in a shaking incubator at 220 rpm. A 20 ul aliquot of the overnight culture was added to 5 ml BHI broth and incubated at 37°C until an OD600 of 0.8 was reached. RNA was extracted from the cultures in the exponential growth phase (OD600 0.8) using PureLink™ RNA Mini Kit (Invitrogen, USA) according to the manufacturer’s instructions. RNA concentration and purity were assessed using a DU 800 Nucleic Acid Spectrometer (Beckman Coulter, USA). The extracts were adjusted to perform rigorous DNase treatments using Deoxyribonuclease I (Invitrogen, USA) in accordance with the manufacturer’s instructions. RNA was reverse transcribed using the RevertAid™ First Strand cDNA Synthesis Kit (Fermentas,Canada). The cDNA was stored at -20°C and prepared for reverse transcription (RT) PCR detection. Primers for the detection of *lukS* mRNA were designed using Primer Premier 5.0 software. The specificity of the primers was assessed by performing a similarity search by BLAST program (Blastn) available in NCBI (http://blast.ncbi.nlm.nih.gov/Blast). The gene *gyrB* was selected to be the endogenous control gene. The primers for detection of *gyrB* mRNA was described previously[15]. The reaction mixture (final volume of 20µl) consisted of 10µl Platinum ® SYBR® Green qPCR SuperMix-UDG, 0.4µl primers (200nM final concentration), 0.4µl ROX Reference Dye, 2µl cDNA, and DEPC water for a final volume of 20µl. The thermocycling program consisted of one hold at 50°C for 2 min and one hold at 95°C for 2 min, followed by 40 cycles of 15 s at 95°C and 30 s at 60°C. Melting curve data were performed at 60°C to 95°C as per guidelines for the ABI 7000 quantitative PCR instrument. Each sample was tested on three separate occasions, and mean values of repeated results were used for further analysis with ABI 7000 software. 

### Multilocus sequence typing (MLST)

MLST analysis by PCR amplification and sequencing of the seven housekeeping genes (*arc*, *aroE*, *glpF*, *gmk*, *pta*, *tpi*, and *yqiL*) of all tested isolates was performed as described previously [16]. The sequences were compared with the existing sequences available on the MLST website for *S. aureus* (http://saureus.mlst.net), and STs were assigned according to the allelic profiles. MLST clonal complexes (CCs) were determined using the program eBURST (based upon related sequence types) available on the MLST website for *S. aureus* (http://saureus.mlst.net). 

### Analysis of data

The data were analyzed using SPSS 17.0 statistic software. Given unequal distribution of *lukS* mRNA, the comparison of two groups was analyzed using median *lukS* mRNA values and the Wilcoxon rank-sum test. Statistical significance was defined by a *p*-value < 0.05. 

## Results

### Characterization of *S. aureus* isolates

Among 96 PVL-positive *S. aureus* isolates, 52 (54.2%) were classified as MRSA. The genotypic profiles are listed in Table 1. A total of 36 STs were identified, with ST88 accounting for 19.8% (19/96), followed by ST239 (8.3%, 8/96), ST59 (7.3%, 7/96), ST188 (5.2%, 5/96) and ST630 (4.2%, 4/96). The remaining STs accounted for ≤ 3 isolates each. Major STs representing MRSA and MSSA isolates, respectively, were as follows: ST88, 9 and 10; ST239, 3 and 5; ST59, 4 and 3; ST188, 5 and 0; ST630, 3 and 1. 36 STs belonged to 10 CCs. CC5, CC88, CC8 and CC59 accounted for 26.0%(25/96), 21.9% (21/96), 12.5% (12/96) and 9.4% (9/96), respectively. 

**Table 1:** Genotypes of *S. aureus* clinical isolates.

CC (No.)^a^	ST(No)	MRSA (No.)	MSSA (No.)	SSTI (No.)	BSI (No.)	Adults (No.)	Children (No.)	HA isolates(No.)	CA isolates (No.)	Median^b^	Mean value and standard deviation
88(21)	88(19)	9	10	9	8	13	6	13	4	0.933	1.536±1.970
	1219(2)		2		1	1	1	2			
8(12)	239(8)	3	5	6	1	7	1	7		0.052	1.514±3.212
	630(4)	3	1		4	4		3	1		
59(9)	2207(2)		2	1	1	2		2		1.025	1.211±1.322
	59(7)	4	3		6	5	2	2	3		
5(25)	5(3)	1	2	3		2	1	3		1.680	2.210±3.050
	6(3)	1	2		2	1	2	2	1		
	15(3)	1	2		2	3		3			
	25(3)	2	1	2	1	1	2		3		
	72(1)	1		1			1				
	774(1)	1			1	1		1			
	199(1)	1			1		1				
	1018(2)	2		2		1	1		2		
	1995(1)		1		1		1		1		
	2104(1)		1		1		1		1		
	2114(1)	1			1	1		1			
	2204(2)		2		1	2			2		
	2212(1)		1		1		1	1			
	2201(1)	1			1		1	1			
	2206(1)		1		1		1	1			
121(7)	2213(1)	1			1		1		1	NC	NC
	2209(1)	1			1		1		1		
	120(2)	2			2	1	1		2		
	1301(3)	1	2	2	1	3			2		
7(6)	7(3)	2	1	3		3		2	1	NC	NC
	943(3)	1	2		2	2	1	3			
1(5)	188(5)	5			5	5		4	1		
398(4)	398(3)	2	1	2	1	3		2	1	NC	NC
	2199(1)	1			1	1		1			
182(2)	944(2)		2		2	2		1		NC	NC
Singletons(5)	1349(1)	1		1		1		1		NC	NC
	1357(1)	1		1		1		1			
	1732(1)	1			1	1			1		
	2203(1)	1			1		1	1			
	2202(1)	1			1		1		1		

^a^ CC, clonal complex.

^b^ NC, not count.

### The expression levels of *lukS-PV* mRNA associate with specific clinical scenarios

Variation of *lukS-PV* mRNA expression levels between the MLST CCs of tested *S. aureus* isolates was identified. However, significant differences were not found between the MLST CCs (*p*>0.05). The expression levels for *lukS-PV* mRNA are provided in Table 2. Among the 33 SSTI isolates, 12 (36.4%) were MRSA, and among the 54 BSI isolates, 33 (61.1%) were MRSA. *lukS-PV* mRNA expression among SSTI isolates was 2.964-fold higher than for BSI isolates. There was significant difference between the expression levels of *lukS-PV* mRNA amongSSTI and BSI isolates (*p* <0.05). 

**Table 2:** Expression levels of *lukS*mRNA among *S. aureus* clinical isolates.

Group	No. of isolates	Median	Minimum value	Maximum value	Mean value and standard deviation
SSTIs isolates	33	1.500^*^	0.000037	14.470	2.789 ± 3.468
BSI isolates	54	0.818	0.000001	3.042	0.941 ± 0.899
Isolates from children	29	1.292^*^	0.000003	5.296	1.565 ± 1.152
Isolates from adults	67	0.540	0.000001	14.470	1.552 ± 2.706
BSI isolates from children	22	1.495^*^	0.000003	3.042	1.726 ± 0.792
BSI isolates from adults	32	0.191	0.000001	1.912	0.400 ± 0.469
CA isolates	28	1.034^*^	0.000037	14.470	2.858 ± 3.823
HA isolates	58	0.536	0.000001	10.891	1.292 ± 2.119

^*^ p<0.05

 41 (61.2%) of 67 isolates from adult patients were MRSA, whereas 11 (37.9%) of 29 tested isolates from children were MRSA. Compared with the isolates from adult patients, **t**he expression level of *lukS-PV* mRNA among isolates from children was significantly higher (*p* <0.05). More specifically, the expression level of *lukS-PV* mRNA among BSI isolates from children was significantly higher than that among BSI isolates from adults (*p* < 0.01). 

 The frequencies of MRSA among 28 CA and 58 HA isolates were 46.4% (13/28) and 58.6% (34/58). The expression level of *lukS-PV* mRNA among CA isolates was 2.212-fold higher than that among HA isolates. There was significant difference between the expression levels of *lukS-PV* mRNA among CA and HA isolates (*p* <0.05)

## Discussion

PVL is a pore-forming, bi-component toxin secreted by *S. aureus* isolates epidemiologically associated with severe infections, including necrotizing pneumonia and SSTIs[1]. PVL lyses polymorphonuclear leukocytes (PMN) of human and rabbit origin, but not those of mice and monkey origin[6]. Higher PVL concentrations cause PMN lysis, and lysed PMNs release inflammatory factors to damage tissues, while lower concentrations of PVL mediate a novel pathway of PMN apoptosis by directly binding to mitochondrial membranes [17]. The importance of PVL in pathogenesis for *S. aureus* SSTIs and necrotizing pneumonia, and MRSA infections, is controversial[5,9,18]. Conflicting data may relate to the amount of PVL produced by individual strains. One study demonstrated that strains with more PVL production produced larger skin lesions and higher bacterial burdens within the lesions in a murine skin infection model[11]. It is possible that the polymorphisms of DNA sequences in PVL genes may result in changes in the function of the PVL protein, which may help to explain the conflicting results seen in animal studies[1]. Regardless, it may be of interest to development quantitative assays for PVL gene expression to determine their clinical utility in predicting disease severity and treatment response. 

 One previous study reported that *S. aureus* isolates associated with severe infections were confined to a relatively small number of important clones[19]. PVL-positive *S. aureus* isolates in our study showed considerable genetic heterogeneity, with 36 genotypes. We found that the PVL was expressed in all isolates carrying Panton-Valentine leukocidin genes detected by qRT-PCR (data not shown), suggesting that PVL genes are always transcribed when they are present in clinical isolates. One study demonstrated that expression levels of PVL genes varied from strain to strain, with more than 10-fold variance[20]. Another study reported that PVL production in MRSA was variable [21]. A wide range for *lukS*-PV transcript expression was observed among clinical isolates in the present study. PVL production in MRSA was found to be associated with MLST CC [21]. In contrast, significant differences were not found between MLST CCs (*p*>0.05) in the present study. 

*S. aureus* clinical isolates carrying PVL genes are often associated with SSTIs requiring incision and drainage[22–24], and the production of PVL correlated with SSTI severity in the aforementioned *S. aureus* animal model[11]. In our study, the expression level of *lukS* mRNA among SSTI isolates was significantly higher than that among BSI isolates, potentially indicating that quantitative PVL gene expression plays a more important role in *S. aureus* SSTIs than for other clinical presentations. However,  a multinational trial found that PVL was not the primary determinant of outcome in patients with MRSA complicated skin and skin structure infections[8]. Boakes et al. reported that there is no statistical relationship between PVL production and the most severe clinical presentations of PVL-MRSA infection[21]. Given the retrospective nature of our study, we did not have sufficient data extracted from the medical record to determine SSTI disease severity, but prospective studies are now justified to determine whether *lukS* mRNA levels correlate with severity of *S. aureus* SSTI infections. 

Children with invasive PVL-positive *S. aureus* infections incur significantly longer hospital stays than pediatric patients with PVL- negative invasive infections, and PVL is thought to play an important role in pathogenesis of S. aureus infection among children [25,26]. One study also reported a correlation between severity of *S. aureus* infections and the presence of PVL genes among adult patients, but quantitative PVL expression, at either gene or protein levels, was not determined[8]. Our study found that expression level of *lukS-PV* mRNA among BSI isolates from children was significantly greater than for BSI isolates from adult patients, but further prospective studies are required to determine whether quantitative *lukS* expression correlates with disease severity in this scenario. Carriage of PVL genes has been closely associated with infections caused by CA-MRSA, including SSTIs, necrotizing pneumonia, and severe sepsis in numerous epidemiological studies[1,27,28]. In our study, the expression level of *lukS-PV* mRNA for CA isolates was significantly greater than for HA isolates. 

In conclusion, we found that quantitative expression of *lukS-PV* mRNA is greater for *S. aureus* SSTIs versus BSIs, for *S. aureus* isolates from children versus adults (in particular for BSIs), and for CA isolates versus HA isolates. These data justify additional prospective studies to determine whether quantitative *lukS-PV* mRNA expression may prove useful for predicting severity of *S. aureus* infection and/or responses to treatment. 

## References

[1] David MZ, Daum RS (2010) Community-associated methicillin-resistant Staphylococcus aureus: epidemiology and clinical consequences of an emerging epidemic. Clin Microbiol Rev 23: 616-687. doi:10.1128/CMR.00081-09. PubMed: 20610826. 20610826 PMC2901661

[2] Saïd-Salim B, Mathema B, Kreiswirth BN (2003) Community-acquired methicillin-resistant Staphylococcus aureus: an emerging pathogen. Infect Control Hosp Epidemiol 24: 451-455. doi:10.1086/502231. PubMed: 12828324. 12828324

[3] Francis JS, Doherty MC, Lopatin U, Johnston CP, Sinha G et al. (2005) Severe community-onset pneumonia in healthy adults caused by methicillin-resistant Staphylococcus aureus carrying the Panton-Valentine leukocidin genes. Clin Infect Dis 40: 100-107. doi:10.1086/427148. PubMed: 15614698. 15614698

[4] Miller LG, Perdreau-Remington F, Rieg G, Mehdi S, Perlroth J et al. (2005) Necrotizing fasciitis caused by community-associated methicillin-resistant Staphylococcus aureus in Los Angeles. N Engl J Med 352: 1445-1453. doi:10.1056/NEJMoa042683. PubMed: 15814880. 15814880

[5] Labandeira-Rey M, Couzon F, Boisset S, Brown EL, Bes M et al. (2007) Staphylococcus aureus Panton-Valentine leukocidin causes necrotizing pneumonia. Science 315: 1130-1133. doi:10.1126/science.1137165. PubMed: 17234914. 17234914

[6] Löffler B, Hussain M, Grundmeier M, Brück M, Holzinger D et al. (2010) Staphylococcus aureus panton-valentine leukocidin is a very potent cytotoxic factor for human neutrophils. PLoS Pathog 6: e1000715 PubMed: 20072612. 20072612 10.1371/journal.ppat.1000715 PMC2798753

[7] Tong A, Tong SY, Zhang Y, Lamlertthon S, Sharma-Kuinkel BK et al. (2012) Panton-Valentine leukocidin is not the primary determinant of outcome for Staphylococcus aureus skin infections: evaluation from the CANVAS studies. PLOS ONE 7: e37212. doi:10.1371/journal.pone.0037212. PubMed: 22623995. 22623995 PMC3356380

[8] Bae IG, Tonthat GT, Stryjewski ME, Rude TH, Reilly LF et al. (2009) Presence of genes encoding the panton-valentine leukocidin exotoxin is not the primary determinant of outcome in patients with complicated skin and skin structure infections due to methicillin-resistant Staphylococcus aureus: results of a multinational trial. J Clin Microbiol 47: 3952-3957. doi:10.1128/JCM.01643-09. PubMed: 19846653. 19846653 PMC2786648

[9] Voyich JM, Otto M, Mathema B, Braughton KR, Whitney AR et al. (2006) Is Panton-Valentine leukocidin the major virulence determinant in community-associated methicillin-resistant Staphylococcus aureus disease? J Infect Dis 194: 1761-1770. doi:10.1086/509506. PubMed: 17109350. 17109350

[10] Shallcross LJ, Fragaszy E, Johnson AM, Hayward AC (2013) The role of the Panton-Valentine leucocidin toxin in staphylococcal disease: a systematic review and meta-analysis. Lancet Infect Dis 13: 43-54. doi:10.1016/S1473-3099(12)70238-4. PubMed: 23103172. 23103172 PMC3530297

[11] Varshney AK, Martinez LR, Hamilton SM, Bryant AE, Levi MH et al. (2010) Augmented production of Panton-Valentine leukocidin toxin in methicillin-resistant and methicillin-susceptible Staphylococcus aureus is associated with worse outcome in a murine skin infection model. J Infect Dis 201: 92-96. doi:10.1086/648613. PubMed: 19929693. 19929693 PMC4084919

[12] Hamilton SM, Bryant AE, Carroll KC, Lockary V, Ma Y et al. (2007) In vitro production of panton-valentine leukocidin among strains of methicillin-resistant Staphylococcus aureus causing diverse infections. Clin Infect Dis 45: 1550-1558. doi:10.1086/523581. PubMed: 18190315. 18190315

[13] McClure JA, Conly JM, Lau V, Elsayed S, Louie T et al. (2006) Novel multiplex PCR assay for detection of the staphylococcal virulence marker Panton-Valentine leukocidin genes and simultaneous discrimination of methicillin-susceptible from -resistant staphylococci. J Clin Microbiol 44: 1141-1144. doi:10.1128/JCM.44.3.1141-1144.2006. PubMed: 16517915. 16517915 PMC1393128

[14] Yu F, Yang L, Pan J, Chen C, Du J et al. (2011) Prevalence of virulence genes among invasive and colonising Staphylococcus aureus isolates. J Hosp Infect 77: 89-91. doi:10.1016/j.jhin.2010.07.019. PubMed: 21030110. 21030110

[15] Duquenne M, Fleurot I, Aigle M, Darrigo C, Borezée-Durant E et al. (2010) Tool for quantification of staphylococcal enterotoxin gene expression in cheese. Appl Environ Microbiol 76: 1367-1374. doi:10.1128/AEM.01736-09. PubMed: 20061456. 20061456 PMC2832361

[16] Enright MC, Day NP, Davies CE, Peacock SJ, Spratt BG (2000) Multilocus sequence typing for characterization of methicillin-resistant and methicillin-susceptible clones of Staphylococcus aureus. J Clin Microbiol 38: 1008-1015. PubMed: 10698988. 10698988 10.1128/jcm.38.3.1008-1015.2000 PMC86325

[17] Genestier AL, Michallet MC, Prévost G, Bellot G, Chalabreysse L et al. (2005) Staphylococcus aureus Panton-Valentine leukocidin directly targets mitochondria and induces Bax-independent apoptosis of human neutrophils. J Clin Invest 115: 3117-3127. doi:10.1172/JCI22684. PubMed: 16276417. 16276417 PMC1265849

[18] Bubeck Wardenburg J, Bae T, Otto M, Deleo FR, Schneewind O (2007) Poring over pores: alpha-hemolysin and Panton-Valentine leukocidin in Staphylococcus aureus pneumonia. Nat Med 13: 1405-1406. doi:10.1038/nm1207-1405. PubMed: 18064027. 18064027

[19] Enright MC, Robinson DA, Randle G, Feil EJ, Grundmann H et al. (2002) The evolutionary history of methicillin-resistant Staphylococcus aureus (MRSA). Proc Natl Acad Sci U S A 99: 7687-7692. doi:10.1073/pnas.122108599. PubMed: 12032344. 12032344 PMC124322

[20] Saïd-Salim B, Mathema B, Braughton K, Davis S, Sinsimer D et al. (2005) Differential distribution and expression of Panton-Valentine leucocidin among community-acquired methicillin-resistant Staphylococcus aureus strains. J Clin Microbiol 43: 3373-3379. doi:10.1128/JCM.43.7.3373-3379.2005. PubMed: 16000462. 16000462 PMC1169154

[21] Boakes E, Kearns AM, Badiou C, Lina G, Hill RL et al. (2012) Do differences in Panton-Valentine leukocidin production among international methicillin-resistant Staphylococcus aureus clones affect disease presentation and severity? J Clin Microbiol 50: 1773-1776. doi:10.1128/JCM.06421-11. PubMed: 22205815. 22205815 PMC3347095

[22] Crawford SE, David MZ, Glikman D, King KJ, Boyle-Vavra S et al. (2009) Clinical importance of purulence in methicillin-resistant Staphylococcus aureus skin and soft tissue infections. J Am Board Fam Med 22: 647-654. doi:10.3122/jabfm.2009.06.090025. PubMed: 19897693. 19897693

[23] Zanger P, Nurjadi D, Schleucher R, Scherbaum H, Wolz C et al. (2012) Import and spread of Panton-Valentine Leukocidin-positive Staphylococcus aureus through nasal carriage and skin infections in travelers returning from the tropics and subtropics. Clin Infect Dis 54: 483-492. doi:10.1093/cid/cir822. PubMed: 22104084. 22104084

[24] Daskalaki M, Rojo P, Marin-Ferrer M, Barrios M, Otero JR et al. (2010) Panton-Valentine leukocidin-positive Staphylococcus aureus skin and soft tissue infections among children in an emergency department in Madrid, Spain. Clin Microbiol Infect 16: 74-77. doi:10.1111/j.1469-0691.2009.02830.x. PubMed: 19519839. 19519839

[25] Cupane L, Pugacova N, Berzina D, Cauce V, Gardovska D et al. (2012) Patients with Panton-Valentine leukocidin positive Staphylococcus aureus infections run an increased risk of longer hospitalisation. Int J Mol Epidemiol Genet 3: 48-55. PubMed: 22493751. 22493751 PMC3316447

[26] Chiu YK, Lo WT, Wang CC (2012) Risk factors and molecular analysis of Panton-Valentine leukocidin-positive methicillin-susceptible Staphylococcus aureus colonization and infection in children. J Microbiol Immunol Infect 45: 208-213. doi:10.1016/j.jmii.2011.11.011. PubMed: 22575426. 22575426

[27] Boussaud V, Parrot A, Mayaud C, Wislez M, Antoine M et al. (2003) Life-threatening hemoptysis in adults with community-acquired pneumonia due to Panton-Valentine leukocidin-secreting Staphylococcus aureus. Intensive Care Med 29: 1840-1843. doi:10.1007/s00134-003-1918-5. PubMed: 12904849. 12904849 PMC7095030

[28] Garnier F, Tristan A, François B, Etienne J, Delage-Corre M et al. (2006) Pneumonia and new methicillin-resistant Staphylococcus aureus clone. Emerg Infect Dis 12: 498-500. doi:10.3201/eid1203.051040. PubMed: 16704793. 16704793 PMC3291452

